# A quantitative model to ensure capacity sufficient for timely access to care in a remote patient monitoring program

**DOI:** 10.1002/edm2.435

**Published:** 2023-06-21

**Authors:** Annie Chang, Michael Z. Gao, Johannes O. Ferstad, Paul Dupenloup, Dessi P. Zaharieva, David M. Maahs, Priya Prahalad, Ramesh Johari, David Scheinker

**Affiliations:** ^1^ Icahn School of Medicine at Mount Sinai New York New York USA; ^2^ Department of Management Science and Engineering Stanford University Stanford California USA; ^3^ Department of Paediatric, Division of Paediatric Endocrinology Stanford University Stanford California USA; ^4^ Stanford Diabetes Research Centre Stanford University Stanford California USA

**Keywords:** capacity planning, diabetes technology, paediatric endocrinology, remote patient monitoring, type 1 diabetes

## Abstract

**Introduction:**

Algorithm‐enabled remote patient monitoring (RPM) programs pose novel operational challenges. For clinics developing and deploying such programs, no standardized model is available to ensure capacity sufficient for timely access to care. We developed a flexible model and interactive dashboard of capacity planning for whole‐population RPM‐based care for T1D.

**Methods:**

Data were gathered from a weekly RPM program for 277 paediatric patients with T1D at a paediatric academic medical centre. Through the analysis of 2 years of observational operational data and iterative interviews with the care team, we identified the primary operational, population, and workforce metrics that drive demand for care providers. Based on these metrics, an interactive model was designed to facilitate capacity planning and deployed as a dashboard.

**Results:**

The primary population‐level drivers of demand are the number of patients in the program, the rate at which patients enrol and graduate from the program, and the average frequency at which patients require a review of their data. The primary modifiable clinic‐level drivers of capacity are the number of care providers, the time required to review patient data and contact a patient, and the number of hours each provider allocates to the program each week. At the institution studied, the model identified a variety of practical operational approaches to better match the demand for patient care.

**Conclusion:**

We designed a generalizable, systematic model for capacity planning for a paediatric endocrinology clinic providing RPM for T1D. We deployed this model as an interactive dashboard and used it to facilitate expansion of a novel care program (4 T Study) for newly diagnosed patients with T1D. This model may facilitate the systematic design of RPM‐based care programs.

## Graphical abstract

We developed a flexible model and interactive dashboard of capacity planning for whole‐population RPM‐based care for T1D. Using this model, we facilitated expansion of a novel care program for newly diagnosed patients with T1D.

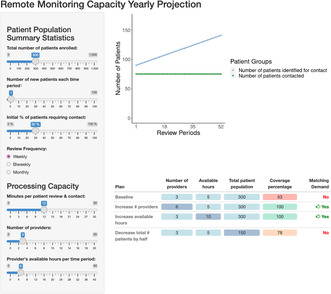

## 1 INTRODUCTION

Telemedicine is an important tool in diabetes management, particularly with the increased adoption of remote patient care during the COVID‐19 pandemic.^1^, ^2^ Remote patient monitoring (RPM), or the use of technologies to monitor medical data, has been associated with significant improvement in glycaemic control.^3^ Continuous glucose monitoring (CGM) makes available detailed information about glucose levels and trends to inform treatment decisions.^4^ Programs providing RPM for paediatric populations affected by diabetes use decision‐support software to process the thousands of data points generated by CGM and identify patients with deteriorating glucose management.^5^, ^6^ We previously showed that the use of a tool, Timely Interventions for Diabetes Excellence (TIDE), which analyses CGM data and provides alerts to facilitate the remote analysis of CGM data, was associated with improved glucose management for patients with type 1 diabetes (T1D).^9^ Open‐source, whole‐population platforms are available to prioritize patients for review by Certified Diabetes Care and Education Specialists (CDCESs) based on their CGM data.^7^, ^8^ By automating the processing of CGM data, this kind of technology has been associated with reduced time spent by CDCES on patient review and an increase in clinic capacity.^9^ A systematic review of RPM for T1D found that the most successful programs used asynchronous messaging.^10^ Numerous hospitals and clinics have made attempts to deploy RPM programs with a variety of technical similar tools, but significant progress has yet to be made in improving algorithm‐based RPM.^11^, ^12^

**FIGURE 1:**
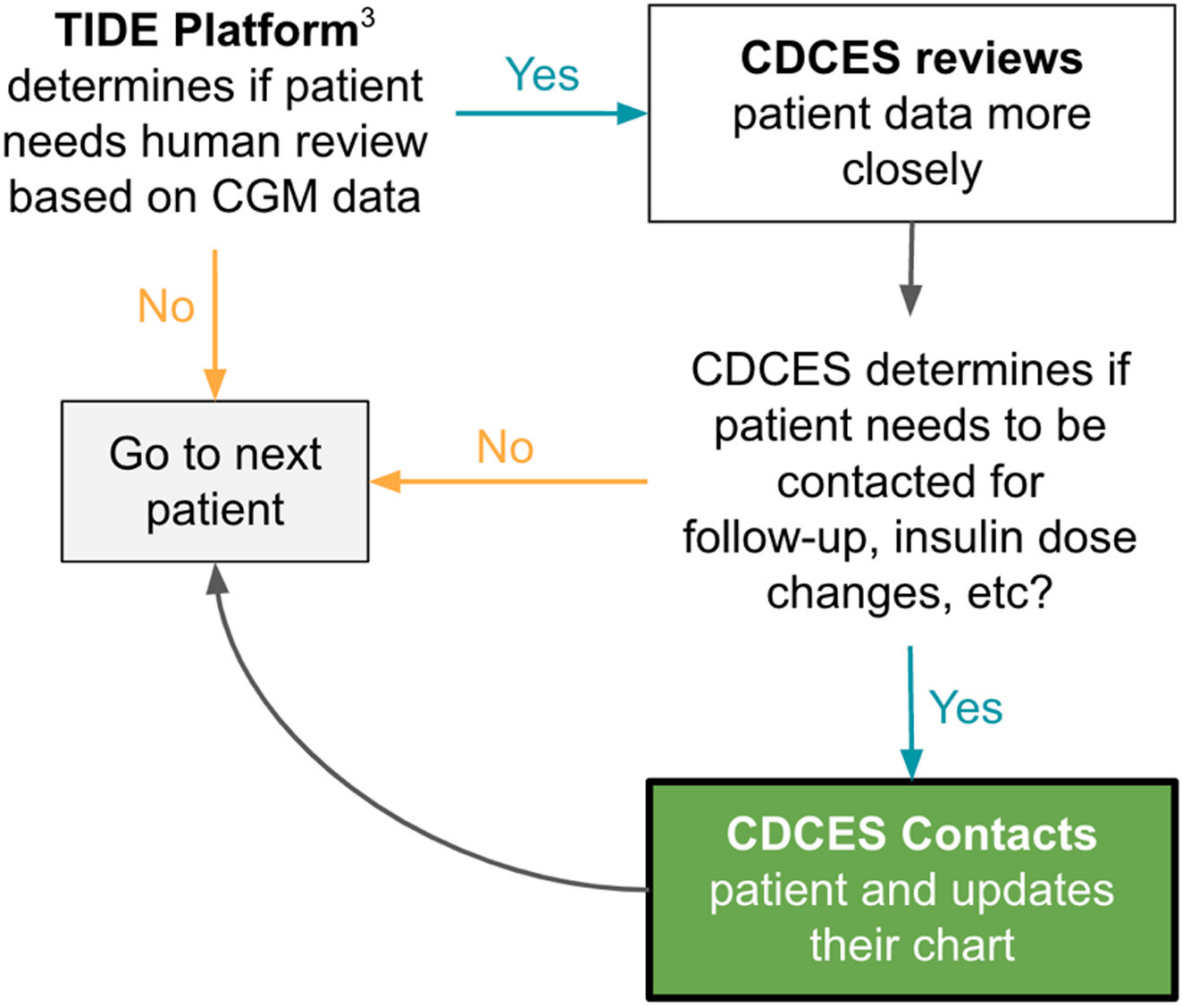
Figure of TIDE Workflow.

Many countries allow the use of CGM in children from manifestation, presenting an opportunity for high‐frequency therapy adjustment from the onset.^13^ However, not all children require therapy adjustment with the same frequency, depending on the course with or without remission and many other factors. As a rule, it is not possible to constantly keep track of the patients' data. Therefore, automatic analysis software for CGM data offers the chance to pre‐filter patients who need therapy adjustment now. The TIDE tool was tested in the context of the Dexcom server. TIDE, or any other algorithm‐enabled tool, can provide relevant patient data to care providers based on which they can choose what course of action to take (Figure 1).

Operational planning poses a significant challenge to establishing a technology‐enabled RPM program. The central role of an algorithm‐driven approach to identifying patients in need of care differs from traditional workflows and requires clinics to estimate the level of care‐provider capacity necessary to meet the needs of the population. Relatively few resources are available to facilitate data‐driven capacity planning for the establishment of whole‐population telemedicine‐based care in paediatric endocrinology clinics. This operational planning tool presented in this paper is a companion tool to the financial model previously developed by our team to aid in the management of telemedicine‐based diabetes care clinics.^14^ Here, we describe the creation of a model to aid in capacity planning and an interactive dashboard as a specific instance of this model. We sought to design and evaluate a formal capacity planning model in order to reduce barriers to implementing novel interventions such as RPM.

## 2 METHODS

*Setting*: This study took place at a paediatric T1D clinic at an academic medical centre as part of the Teamwork, Targets, Technology and Tight Control (4 T) Study and the CGM Time in Range Program at Stanford (CGM TIPS), programs that provide RPM based on data from CGM. Both programs were approved by The Stanford University Institutional Review Board and participants or their legal guardians gave informed consent. The details of the study protocols, the patient populations and the development of the technology used have been reported in detail.^15^, ^16^ All participants in the 4 T Study and its predecessor, the 4 T Pilot Study, were started on a CGM system (Dexcom G6, Dexcom Inc) within the first month of diabetes diagnosis.^16^, ^17^ Participants in the 4 T Pilot and the 4 T Study 1 were between the ages of 1 and 21, were newly diagnosed with T1D over the past 30 days and were willing to operate an Apple device whose data was shared with the T1D clinic. Weekly remote monitoring of CGM data was provided for newly diagnosed T1D patients beginning in March 2019. Participants in the CGM TIPS study were enrolled between July 2020 and April 2022, and the study is currently ongoing.^18^, ^19^ The number of participants continues to grow with enrolment throughout the study. These participants were covered by public insurance, diagnosed with T1D of any duration and were willing to operate an Apple device whose data was shared with the T1D clinic.

The RPM program has four primary components: (1) each patient wears a CGM from which data are uploaded to the manufacturer's server via the patient's mobile device from which they are available to be downloaded by the clinic, (2) CDCESs review CGM data weekly for the 4 T population and monthly for the TIPS population, (3) a platform automatically downloads the data and flags patients for review based on a combination of American Diabetes Association consensus metrics and algorithms,^7^, ^9^, ^15^ and (4) CDCESs review flagged patient data, send patients secure messages through the electronic medical record (EMR), and update the patient chart in the EMR.

*General setting*: The capacity planning model avoids assumptions specific to the use of continuous glucose monitors or the workflows or population of the study institution. It is designed for any RPM program that has the same four primary components: (1) a population of patients about whom data are available remotely to care providers, (2) a fixed cadence at which care providers dedicate time to RPM‐based patient care, for example 1 h every Friday, (3) a tool that analyses patient data to *flag* some of the patients for review by care providers and (4) and care providers (e.g. a CDCES or physician) that *review* the flagged patient data and based on the data select patients to *contact* to provide guidance.

In the TIDE program, flagged patients are those identified by the algorithm as potentially needing attention due to certain criteria being met, such as spending more than 5% of the time with glucose levels below 70 mg/dL. The criteria for review and outreach may vary depending on the study or clinic's specific guidelines. In our experience, a small subset of flagged patients actually end up not requiring contact because, upon review, the provider may determine that no immediate action is needed or that the patient is already on an appropriate course of action. This is an important aspect for clinics to consider when estimating the percentage of patients requiring contact, as it may significantly affect the model's estimates.

To limit the number of contacts that patients receive and the amount of provider time required, the thresholds for patient contact can be set based on historical patient data in order to limit the percentage of patients flagged to an appropriate level.^10^ For clinics with significantly higher capacity or populations with different needs or preferences, a higher percentage of patients may be contacted each review period, and this model allows the clinic to plan accordingly.

As of April 2022, TIDE is being used in the 4 T Pilot (*n* = 135), 4 T Study 1 (*n* = 133), and CGM TIPs (*n* = 94) studies at Stanford. Three CDCESs conducted remote monitoring for the 277 patients enrolled in these three studies, whose demographics are depicted in Table 1. Remote monitoring was conducted on a weekly basis for 4 T and Pilot 4 T participants and monthly for TIPS participants. Each of the three CDCESs conducted patient monitoring for 4 h per week, and the estimated amount of time spent per patient review was 10 min.

**TABLE 1:** Participants demographics for the 4 T Study, 4 T Pilot study, and CGM TIPS Study. Data reported as mean (SD) or percent (%).

	Study		
	4 T Study 1	4 T Pilot	TIPS
Participants, N	133	135	94
Age, Mean (Range)	10.9 (1.0–17.9)	9.7 (6.7–12.7)	14.6 (11.6–17.4)
Sex			
Female, % (n)	44%, (59)	53%, (71)	49%, (46)
Male, % (n)	56%, (74)	47.4%, (64)	51%, (48)
Self‐identified race			
White, % (n)	38%, (50)	37%, (50)	23%, (22)
Hispanic, % (n)	18% (24)	19% (25)	48% (45)
Asian, % (n)	8% (11)	13%, (17)	3%, (3)
Black, % (n)	1%, (1)	0%, (0)	5%, (5)
Other, % (n)	29%, (38)	8%, (11)	55%, (46)
Not stated or unknown, % (n)	21%, (28)	23.7%, (32)	13%, (12)
Primary language			
English, % (n)	84%, (112)	87%, (117)	65%, (61)
Spanish, % (n)	14%, (19)	13.3%, (18)	32%, (30)
Other or unknown, % (n)	9%, (12)	0%, (0)	3.2%, (3)
Insurance type			
Private, % (n)	35%, (47)	77%, (104)	0%, (0)
Public, % (n)	62%, (83)	23%, (31)	100%, (83)
Both, % (n)	2%, (2)	0%, (0)	0%, (0)

### 2.1 Design

This study consisted of three phases.

The first phase was a retrospective analysis of patient CGM data, messages sent from CDCESs to patients, and data gathered in previous studies of the time required by CDCESs to review patient data and send messages.^7^, ^9^ Using data from January 2021, we estimated the amount of time each provider spent in each review period and the breakdown of the time per patient and per activity, that is to review data and to send a message to the patient.

The second phase was the development of a model of CDCES capacity and the revision of the model based on feedback from stakeholders and user testing. We interviewed stakeholders including paediatric endocrinologists, hospital administrators and data analysts to determine clinical and operational variables relevant to telemedicine‐based diabetes care to be incorporated into the dashboard.

We created an operational planning model to calculate the coverage percentage and demand‐capacity matching for patients in the T1D clinic. In the baseline scenario, values for number of enrolled patients, number of new patients, initial percentage of patients requiring contact, review frequency, review capacity, number of providers and provider availability were set based on the parameters at the T1D clinic as of March 2022. Definitions of these and related terms are in Table 2.

**TABLE 2:** Key terms.

Term	Definition
T1D	type 1 diabetes
CDCES	Certified Diabetes Care and Education Specialist; health professional with comprehensive knowledge in diabetes prevention and management
CGM	continuous glucose monitoring; wearable technology that measures glucoses every 1–15 minutes
RPM	remote patient monitoring; the process of using connected technology to provide care to patients in their own homes
TIDE	Timely Interventions for Diabetes Excellence; A tool that analyzes CGM data and provides alerts to facilitate the remote analysis of CGM data
4 T Study	Teamwork, Targets, Technology, and Tight Control Study; A novel care program started at Stanford University for newly diagnosed paediatric patients with T1D
Pilot 4 T Study	Pilot version of the 4 T Study; conducted between May 24, 2014 and December 31, 2016.
TIPS	Time in Range Program at Stanford; This program initiates CGM in youth with public insurance and provides RPM. Started enrollment July 2020 and the study is ongoing.
Review frequency	How frequently a CDCES reviews the dashboard (e.g., weekly, biweekly, monthly)
Provider availability	The number of providers that are available to review the dashboard in the RPM
Flagged patient	Patients who have been noted to have too many lows, drop in TIR or not meeting target. Flagged patients are contacted unless the patient had an appointment that week or if providers review the data and determine that no immediate action is needed.
Contacted patient	Contacted patients are flagged patients that CDCESs call or message

**FIGURE 2:**
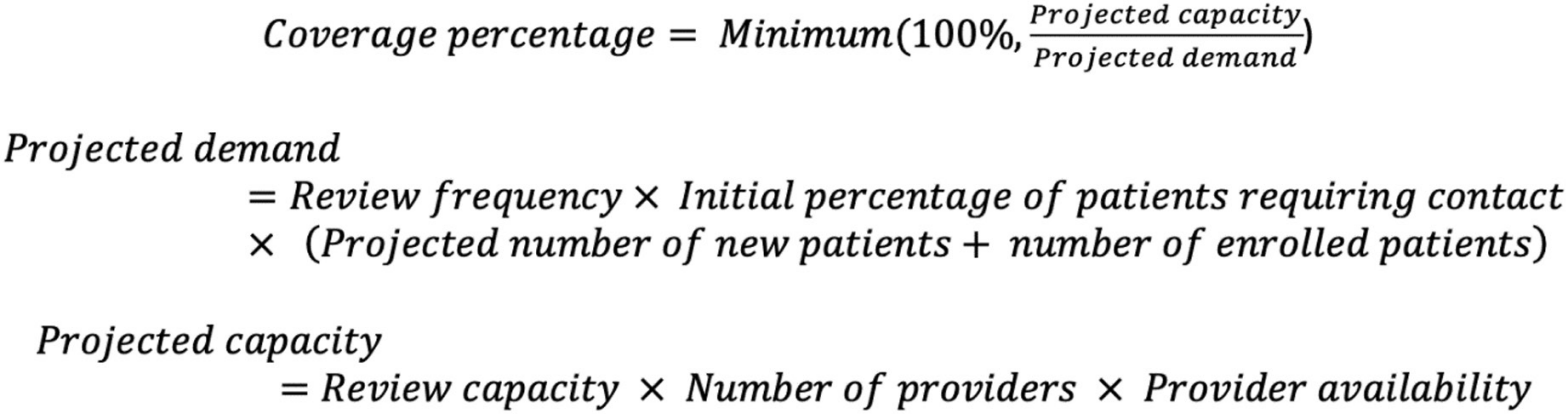
Equations used to make dashboard projections.

We calculated the coverage percentage by dividing the projected demand by the projected capacity. To compute the projected demand, we multiplied three terms together: the review frequency, initial percentage of patients requiring contact and the sum of the projected number of new patients and the number of enrolled patients. To compute our projected capacity, we multiplied together the capacity‐related parameters: review capacity, number of providers and the provider availability. Equations used to make the dashboard projections are displayed in Figure 2.

During the third phase of the evaluation of the projections relative to historical data, we analysed historical data to investigate the coverage percentage of the T1D clinic. While we did not have access to the exact number of patients requiring contact, the historical data included the weekly number of patients flagged for review and the weekly number of patients contacted, although not all patients flagged for review required contact. In the third phase, we evaluated the model with data from the clinic where it was developed. We projected the expected number of patients flagged, reviewed and contacted for January 2022, and compared the projected data to actual data for January 2022. Dataset processing and cleaning were conducted using the *dplyr* package of R Version 1.3.1093.^20^ The capacity planning dashboard was designed using *RShiny*, an open‐source R package for building web applications.^21^

This model was operationalized with an interactive dashboard, users tested the dashboard (CDCESs and physicians) at the study institution, Lucile Packard Children's Hospital and at another paediatric academic medical centre, Children's Mercy Hospital. The inputs are the data points identified as driving provider time use as well as the data on the patient population that determine the need for provider time, for example the number of patients in the population. The number of patients identified for contact was defined as the percentage of patients who need contact multiplied by the number of patients in the program. The CDCES capacity was defined as the number of minutes per review period that each CDCES had available to review patient data and send messages to patients.

Flagged patients are those identified by the algorithm as potentially needing attention due to certain criteria being met (e.g., glucose levels outside a specified range). However, not all flagged patients will ultimately require contact, as a provider may review the data and determine that no immediate action is needed. In our experience, the distinction between flagged patients and those requiring contact is minimal, as our model is designed to accurately identify patients in need of intervention. The demand was measured as minutes required for CDCESs to review patient data and send messages to those patients who meet criteria for contact.

The primary output was the calculated capacity of the care team as a percentage of the time required to review and contact all of the patients identified as needing review and contact. A secondary output was a table of potential modifications, to care provider capacity or the patient population, sufficient to ensure that the calculated capacity meets or exceeds patient demand.

The model was evaluated by comparing the predicted and actual number of patients flagged and contacted in the clinic. We used 52 review periods to determine the number of patients flagged and the number of patients contacted. For historical data, we used data on patient contacts from 2020 to 2022 to identify the number of patients shown, flagged, and contacted in the TIDE program. Model projections were then compared with the actual number of patients flagged and contacted in January 2022.

## 3 RESULTS

**FIGURE 3:**
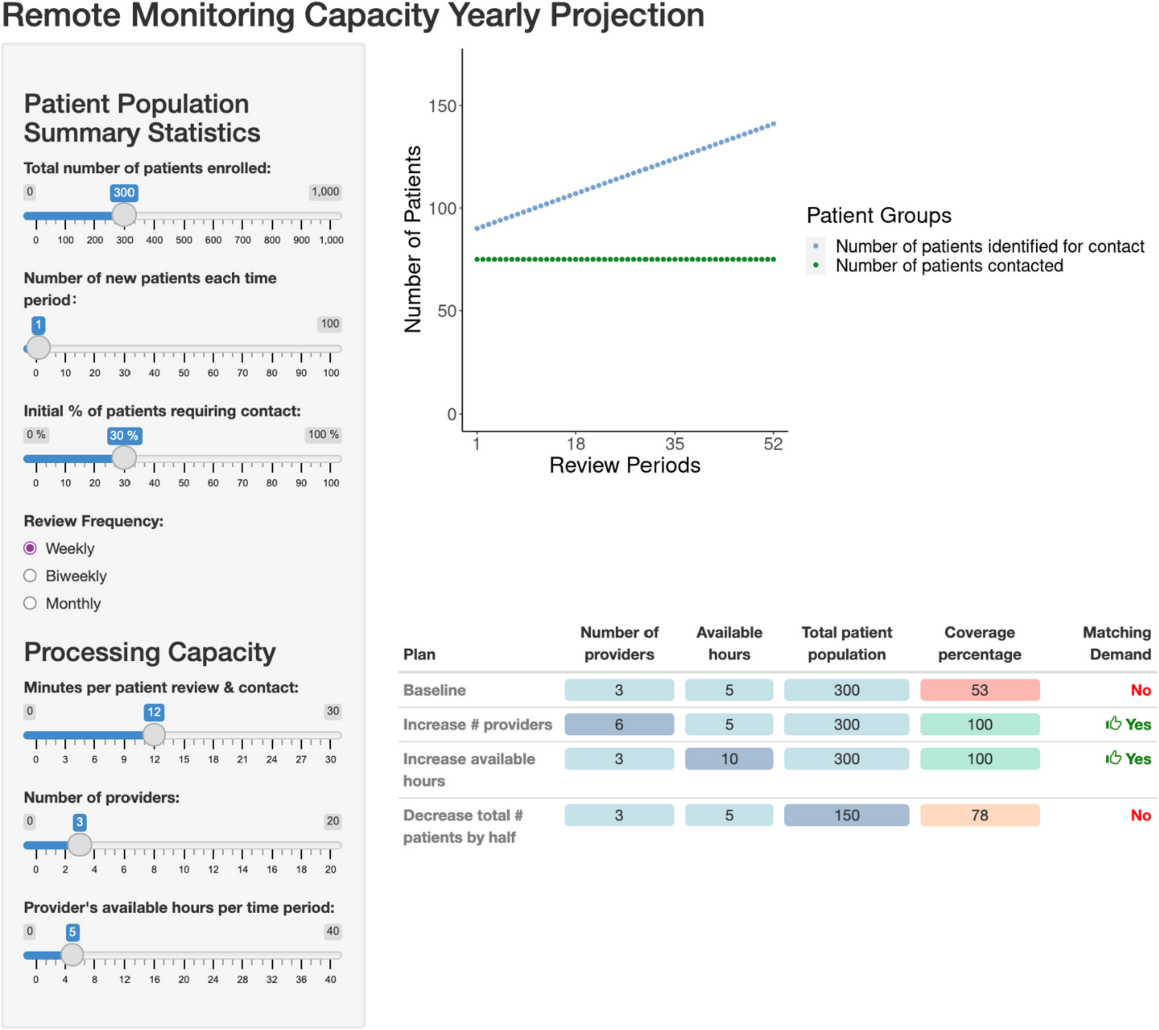
Interface for the capacity planning dashboard. Input parameters for patient population and processing capacity can be modified to project the coverage percentage in the T1D clinic.

The dashboard provides an interface for users to compare their clinic capacity with patient need by varying input parameters and seeing model outputs update dynamically (Figure 3). The dashboard includes three alternative capacity plans that calculates coverage percentage to determine whether demand matches capacity at varying levels of input parameters. To facilitate use by healthcare providers and administrators, the model is available online (https://surf‐tide.shinyapps.io/capacity_dashboard/). The dashboard allows users to visualize and manipulate the primary population‐level drivers of demand in the program. Several rounds of design were performed based on user feedback to improve the accessibility, simplicity, and interpretability of the dashboard.

Multiple ways to achieve maximum clinic capacity were identified by modifying input parameters in the dashboard. Hiring two additional CDCESs (three to five CDCESs) and increasing the number of CDCES available hours by 2 h per review period (4–6 h) each increased projected clinic coverage percentage by 27% (from 73% to 100%). Reducing average patient review time by 2 min (8–10 min per patient) increased projected clinic coverage percentage by 18% (from 73% to 91%).

**FIGURE 4:**
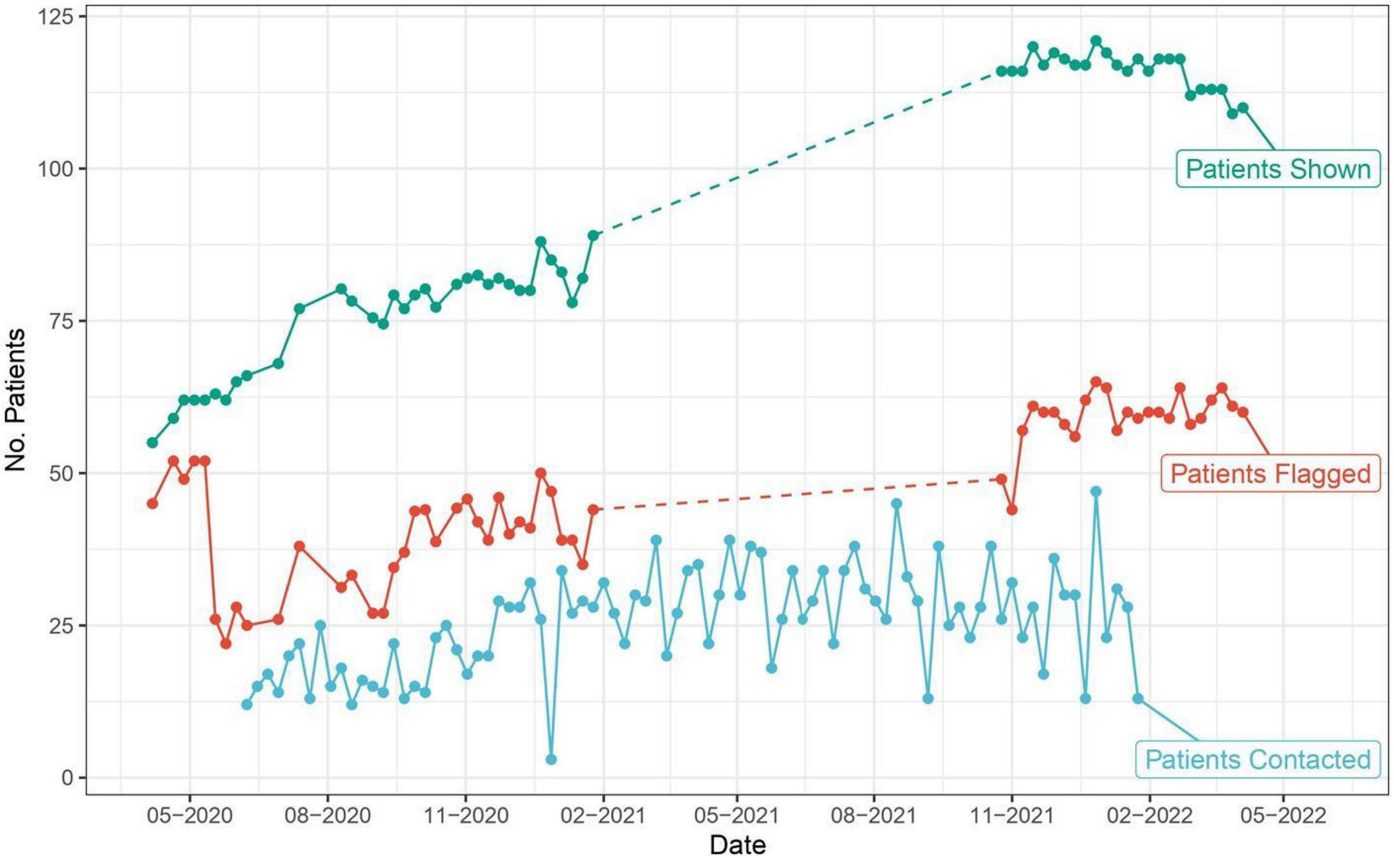
Number of patients shown (green), flagged for review (red), and contacted (blue) from 2020 to 2022 in the TIDE Program. These historical data metrics were compared with dashboard projections to assess the capacity planning dashboard and identify areas for operational improvements.

The ratio between the number of patients flagged for review and number of patients contacted in the T1D was 53.8% by the end of month 3, 43.5% by the end of month 6 and 59.3% by the end of month 9. For reference, the mean of this ratio between March 2020 and April 2022 was 51.8% and the standard deviation was 17.1%. Meanwhile, the number of patients flagged was 26 by the end of month 3, 37 by the end of month 6 and 47 by the end of month 9. The number of patients flagged steadily increased over time and the ratio between the number of patients flagged for review and number of patients contacted did not change significantly (Figure 4).

## 4 DISCUSSION

We designed a generalizable, interactive model for capacity planning that facilitated improved resource allocation to maximize the efficiency of a paediatric endocrinology clinic providing RPM for T1D. The model facilitated the quantitative design of the expansion of RPM to additional patients by identifying the necessary resources. This model has the potential to improve demand‐capacity matching and facilitate efficient care delivery for clinics using or designing RPM programs for diabetes care. One specific application will be in the dissemination of the 4 T Study to other diabetes centres.

We developed a proof‐of‐concept interactive dashboard for capacity planning, which provides a platform to help stakeholders make better operational decisions for population‐level diabetes care. The deployment of the model as an interactive dashboard and its use by clinical leaders revealed several insights. We found that reductions to average patient review time, increases in the number of CDCESs and increases in a CDCES's available hours per time period through the standardization of workflows has the potential to produce substantial improvements to clinic capacity. Hiring additional CDCESs or reducing average patient review time would have corresponded to an estimated 27% and 18% increase, respectively, in clinic capacity based on dashboard projections. One benefit of the scenario analyses in the dashboard is that it highlights differences in capacity as stakeholders make decisions about capacity, rather than absolute levels of capacity for a single choice.

To clarify, our simulation tool can be adapted for various provider types in a single clinic setting by creating a new simulation for each provider type. While the current model relies on averages and may not capture differences in provider efficiency, creating a new simulation for each provider type would enable clinics to evaluate the impact of adding new or experienced providers on their estimates and optimize their resource allocation strategies accordingly.

Regarding privacy issues, TIDE operates within the context of the Dexcom server. The software is not built into the patients' Apple handhelds, and the current data flows from Dexcom to TIDE through HIPAA compliant servers. All patients gave informed consent. TIDE is freely available on Amazon Web Services, and there are several ongoing efforts to expand its use to other clinics. It is conceivable that TIDE software could be integrated into the evaluation at other CGM providers.

We understand that there are limitations in our model. Our model is limited in that we do not consider the variance of patient demand, and that the capacity for each provider can be variable. As a result, our dashboard may not be able to account for short‐term fluctuations in clinic capacity. However, for long‐term capacity planning on the scale of months or years, such average‐based calculations are a useful starting point. Our model is more conservative than reality because it is likely that providers can improve the efficiency of their workflows, such as through training or standardized workflows. The frequency of monitoring and the percentage of participants needing outreach are two factors that are likely to have a complex interaction that can affect the accuracy of our model. Therefore, it is essential to explore this relationship and consider its potential impact when developing our model to ensure a more comprehensive and reliable representation of the phenomenon under study. However, we acknowledge that due to the limitations of the current model, we cannot incorporate this feature at this time and will note this for future study.

The model also does not incorporate changes in the percent of patients requiring contact each period. If this percentage changes significantly from the initial percent, for example as a result of the intervention a lower percent of early patients require contact, the users can rerun the model with updated percentages. This design choice was made to keep the initial model as simple as possible and since running the model takes only a few minutes for an update not likely to be required more frequently than several times a year.

We acknowledge that other clinics might have different ratios of flagged to contacted patients, and we encourage them to carefully consider their own flagged to contacted ratios when using our model. We chose the percentage of patients requiring contact as the model input instead of the percentage flagged because the former is more directly related to the actual workload and impact on clinic capacity. Understanding the ratio of flagged to contacted patients will allow clinics to make more informed decisions about resource allocation and workflow optimization.

It is important to note that the entire concept is based on time‐effective asynchronous consultation, where a physician or diabetes expert evaluates marked reports and changes therapy, putting the information into an electronic patient record. No more time‐consuming phone call or video call is covered here. For true staff scheduling, it would be necessary to calculate those times as well. We will consider adding this aspect to the discussion. Finally, our model is limited because it was calibrated using 1 month of data and tracks the specific characteristics of the patient population in our clinic. However, this also highlights an opportunity in providing a template of building a dashboard based on past data at a single institution that would become more robust with data from more clinics. The scenario analysis from the interactive dashboard is also expected to become stronger with data over longer periods of time.

We acknowledge that advisory software, which even makes suggestions for therapy (pump/MDI) settings, could be the next step in the evolution of our tool. Although such software is not yet available in most countries, the workload might remain the same. Children will use more and more AID pumps in the future, and the data evaluation is much more complex (depending on the software). The more complex the therapy, the more synchronous and thus more time‐consuming, face‐to‐face counselling has to be done. Thus, our tool may be more helpful to those who use only a CGM with MDI for most patients. This point should be considered in future discussions of the context of our tool.

It is also important to acknowledge that the mode of communication used between providers and patients can significantly impact the time spent on RPM and its effectiveness. For EMR messaging, 81% of the messages sent to 4 T patients as part of RPM were read within 7 days. While EMR messaging was the primary mode of communication in the studies used for our model, there are other communication modes, such as phone outreach or text messaging, that may have different impacts on the efficiency of RPM. For instance, we may designate what percentage of the population may require a longer review or contact time via a certain mode of communication, and what percentage will require less time via a different mode of communication, to help clinics better plan their resource allocation for RPM. This could improve the accuracy and utility of our model by providing clinics with a more customized and adaptable framework to meet their specific needs. Recent literature suggests that text‐based communications may offer a more equitable solution, particularly for populations with limited access to healthcare services, such as rural and low‐income communities.^22^ Further studies need to be conducted to understand the responsiveness of individuals to various modalities of RPM communication. Communication should be personalized to the needs of the individual to not introduce disparities.

Ultimately, operational planning can be facilitated and should be done in complement with financial planning. The capacity planning dashboard presented should be used in combination with a financial model to design care delivery that is both operationally efficient and financially sustainable.^14^ As additional metrics, such as those from wearable activity trackers, are integrated into TIDE, our model and the corresponding dashboard can be easily updated to modify review times and population management.^23^

This capacity planning model may serve as a framework for institutions developing tools to assist operational planning. As health systems across the globe increasingly shift towards telemedicine‐based care, the dashboard implementing the model can facilitate quantitative planning to introduce novel RPM‐based care.

## AUTHOR CONTRIBUTIONS

**Michael Z. Gao:** Conceptualization (equal); formal analysis (equal); investigation (equal); software (equal); validation (equal); writing – original draft (supporting); writing – review and editing (supporting). **Johannes O. Ferstad:** Formal analysis (supporting); software (supporting); writing – review and editing (equal). **Paul Dupenloup:** Writing – review and editing (supporting). **Dessi P. Zaharieva:** Writing – review and editing (supporting). **David M. Maahs:** Conceptualization (equal); funding acquisition (equal); investigation (equal); project administration (equal); supervision (equal); writing – review and editing (equal). **Priya Prahalad:** Conceptualization (equal); investigation (equal); project administration (equal); supervision (equal). **Ramesh Johari:** Conceptualization (equal); investigation (equal); project administration (equal); supervision (equal); writing – review and editing (equal).

## FUNDING INFORMATION

This work was supported by the National Institutes of Health (Grant R18DK122422) and Helmsley Charitable Trust (G‐2002‐04251‐ 2).

## CONFLICT OF INTEREST STATEMENT

A.Chang: None. M.Z.Gao: None. J.O.Ferstad: None. P.Dupenloup: None. D.P.Zaharieva: Research Support; Insulet Corporation, International Society for Paediatric and Adolescent Diabetes, Leona M. and Harry B. Helmsley Charitable Trust.

D.M.Maahs: Advisory Panel; Abbott Diabetes, Eli Lilly and Company, Medtronic, Novo Nordisk, Sanofi, Consultant; Aditx Therapeutics, Inc., Biospex. P.Prahalad: None. R.Johari: None. D.Scheinker: Carta Healthcare.

## CONSENT

The Stanford Institutional Review Board approved this protocol and consent (and assent for participants aged 7‐18 years old) was obtained for review of all participants.

Adapted from: The Cost‐Effectiveness of Real‐Time Continuous Glucose Monitoring (RT‐CGM) in Type 2 Diabetes https://journals.sagepub.com/doi/10.1177/1932296816628547;

American Diabetes Association. 7. Diabetes Technology: Standards of Medical Care in Diabetes—2020. Diabetes Care. 2020;43(Supplement 1):S77‐88.

## Data Availability

The data that support the findings of this study are available on request from the senior author, DS. The data are not publicly available due to restrictions that could compromise the privacy of research participants in the remote monitoring program. The model is publicly online: https://surftide.shinyapps.io/capacitydashboard/.
